# From integrative diagnostics to personalised management: a framework combining molecular and spatial immune profiling in NSMP endometrial carcinoma

**DOI:** 10.3389/fimmu.2026.1859445

**Published:** 2026-07-21

**Authors:** Lan Zhong, Liang Song

**Affiliations:** 1 Department of Medical Oncology, Cancer Center, West China Second University Hospital, Sichuan University, Chengdu, Sichuan, China; 2 Key Laboratory of Birth Defects and Related Diseases of Women and Children (Sichuan University), Ministry of Education, Chengdu, Sichuan, China

**Keywords:** endometrial carcinoma, integrative pathology, L1CAM, NSMP, personalised therapy, risk stratification, tumour immune microenvironment

## Abstract

Endometrial carcinoma (EC) of the No Specific Molecular Profile (NSMP) subtype, the largest molecular category, exhibits marked clinical heterogeneity that challenges morphology-based risk assessment. Two complementary biomarker categories have demonstrated independent prognostic value: protein-level markers (L1CAM, oestrogen receptor [ER], and progesterone receptor [PR]) detectable by routine immunohistochemistry and spatially resolved cancer-immune phenotypes (SCIs) that characterize the tumour immune microenvironment. This review synthesises evidence for both, highlighting that they are often considered in isolation. We argue that their systematic integration is essential for a more precise diagnostic framework. We articulate the biological rationale and propose a testable “dual-risk” hypothesis, whereby combined molecular and immune profiling could identify extreme-risk populations. Realising this integrative model requires overcoming standardisation hurdles, but it represents a critical step towards predictive, functionally informed stratification to guide personalised adjuvant therapy decisions, including intensification and de-escalation. We emphasise that this framework remains a testable hypothesis requiring prospective validation before clinical application.

## 1 Introduction: addressing diagnostic challenges through integrative biomarkers

Endometrial carcinoma is the most common gynaecologic malignancy in developed nations. The molecular classification pioneered by The Cancer Genome Atlas (TCGA) has been clinically translated through the Proactive Molecular Risk Classifier for Endometrial Cancer (ProMisE), a tool that stratifies patients based on molecular risk factors. This advancement has significantly improved the accuracy of prognosis and guided more personalised therapeutic strategies (1, 2). Within this paradigm, the No Specific Molecular Profile (NSMP) subtype—defined by the absence of pathogenic POLE mutations, mismatch repair deficiency, and p53 abnormality—constitutes approximately 40–50% of all endometrial cancers (ECs) (3, 4). While generally associated with an intermediate prognosis, NSMP tumours demonstrate remarkably heterogeneous clinical behaviour, encompassing indolent courses and unexpectedly aggressive recurrences (5, 6). This heterogeneity creates a well-recognised “blind spot” in contemporary clinical management, as decisions for adjuvant therapy primarily rely on traditional histopathological risk factors—namely, FIGO stage, tumour grade, histotype, and lymphovascular space invasion (LVSI). Consequently, for many NSMP patients, especially those with early-stage endometrioid tumours of grades 1 and 2, these criteria lack sufficient accuracy in risk stratification, potentially leading to both overtreatment and undertreatment (7).

The recently published PORTEC-4a trial has provided the first prospective, randomised evidence that a molecular integrated risk profile can safely guide adjuvant radiotherapy decisions in high-intermediate risk endometrial cancer (8). In this trial, L1CAM overexpression was formally incorporated as a defining feature of the unfavourable risk profile, triggering pelvic radiotherapy rather than vaginal brachytherapy or observation (8). This marks a pivotal shift: L1CAM is no longer a purely investigational biomarker but has entered clinical decision-making at the highest level of evidence. Concurrently, the RAINBO umbrella program—a series of molecular profile-directed adjuvant trials—is prospectively testing treatment de-escalation and intensification strategies in endometrial cancer. Among its four component trials, the NSMP-ORANGE trial (NCT05255653-3) is a phase III randomised controlled trial specifically evaluating whether, in ER-positive NSMP patients (stage II with LVSI or stage III), adjuvant radiotherapy followed by two years of oral progestin is non-inferior to standard chemoradiation, with the goal of safely reducing treatment-related toxicity.

Therefore, there is an urgent need to refine risk stratification within the NSMP group (6). The field of diagnostic pathology is uniquely positioned to address this by integrating novel, biologically grounded biomarkers into routine practice. Two complementary avenues have shown considerable promise. First, specific protein-level molecular markers, such as the cell adhesion molecule L1CAM and the hormone receptors ER and PR, are readily assessable by standard immunohistochemistry (IHC), and they reflect intrinsic properties of tumour cells. Second, the spatial cancer-immune phenotype (SCI), a histopathological classification describing the topographic relationship between immune cells and carcinoma cells, captures the functional architecture of the tumour immune microenvironment (TIME). This review aims to: (i) synthesise the current evidence supporting the prognostic value of key molecular markers and SCIs specifically in NSMP endometrial carcinoma (NSMP EC); (ii) critically examine the current separation of these biomarker domains in research and diagnostic workflows; (iii) propose a biologically coherent framework for their integration, culminating in a testable “integrative diagnostic subtyping” hypothesis; and (iv) discuss the methodological challenges and future directions for translating this integrative model into predictive diagnostic pathology, ultimately guiding more personalised patient care.

This is a narrative review that synthesises evidence from the literature to develop a conceptual framework for integrating molecular and spatial immune biomarkers in NSMP endometrial cancer. By bridging these currently separate biomarker domains, we aim to lay the groundwork for a novel conceptual approach to NSMP EC diagnosis. Consequently, this review advances a translational framework that synthesizes evidence across molecular and spatial dimensions to convert biological heterogeneity into actionable clinical stratification, thereby directly informing the personalised management of NSMP endometrial carcinoma. Our framework builds upon prior integrated immune-molecular studies by focusing specifically on the NSMP subtype, operationalising categorical spatial phenotypes, and translating the integration into a clinically actionable risk matrix.

## 2 Molecular markers for diagnostic integration: L1CAM and hormone receptors

The search for prognostically relevant molecular markers in NSMP EC has converged on a few key candidates amenable to IHC, facilitating their potential integration into standard pathological reporting.

### 2.1 L1CAM: a marker of high-risk phenotype

L1 cell adhesion molecule (L1CAM) is a transmembrane glycoprotein involved in cell migration, adhesion, and metastasis. Its overexpression in EC is strongly associated with adverse clinicopathological features, including non-endometrioid histology, high grade, and LVSI. Crucially, meta-analyses confirm that L1CAM overexpression is a robust, independent predictor of poor recurrence-free and overall survival in NSMP EC, with hazard ratios (HRs) ranging from 3.42 to 4.31 (2, 9). Recent systematic reviews and meta-analyses focused on the NSMP subtype have consolidated these findings, confirming L1CAM overexpression as a strong predictor of poor survival (pooled HR approximately 4.3) within the NSMP molecular subtype (9, 10). Its prognostic importance may even surpass that of molecular subtype classification in patients with high-risk disease (11). From a diagnostic perspective, L1CAM IHC is feasible and reproducible. A consensus is emerging around using a ≥10% cutoff for positive staining to define high-risk cases (2). Its value lies in identifying a subset of NSMP tumours, including cases otherwise classified as low-grade endometrioid histology, which harbour molecular markers of aggressive disease (12). Thus, L1CAM expression can be viewed as a potential risk modifier, providing critical prognostic information beyond standard tumour grading.

The clinical translation of L1CAM as a treatment-guiding biomarker has now been realised through the PORTEC-4a trial (8). In this phase III non-inferiority trial, L1CAM overexpression—defined alongside p53 abnormality and substantial LVSI—was used to assign patients with unfavourable molecular profiles to pelvic radiotherapy rather than vaginal brachytherapy or observation. This represents the highest level of evidence supporting L1CAM’s role in adjuvant treatment decisions for endometrial cancer, and it strongly validates the inclusion of L1CAM in integrative stratification frameworks such as the one proposed here.

### 2.2 Oestrogen and progesterone receptors: cornerstones of favourable prognosis

The loss of oestrogen receptor (ER) and progesterone receptor (PR) expression is a well-established hallmark of aggressive EC biology. In the context of NSMP tumours, ER negativity is a powerful independent adverse prognostic factor. Meta-analyses demonstrate the prognostic significance of ER status in NSMP tumours. Specifically, ER-positive NSMP tumours have a significantly lower risk of recurrence (HR 0.37) and death (HR 0.22) (9, 13). These estimates are strongly supported by a recent comprehensive meta-analysis, which reported pooled HRs of 0.37 for recurrence and 0.22 for overall survival specifically in NSMP ECs (9). PR loss shows an association with worse outcomes that is similar to, and often correlated with, ER loss. Multiple meta-analyses have demonstrated that higher PR expression is associated with favourable survival in endometrial cancer, and PR positivity predicts response to progestin therapy in advanced and recurrent disease. However, the independent prognostic value of PR specifically within the NSMP subtype is less well characterised than that of ER. Reflecting this uncertainty, the 2025 ESGO–ESTRO–ESP guidelines did not include PR in their risk stratification algorithm, a decision that has been questioned by some investigators who argue that PR has additional prognostic and predictive value and is a cheap, well-established biomarker (13). In our framework, we therefore position PR as a supportive rather than primary stratification marker, with its interpretation considered in conjunction with ER status. Consequently, IHC for ER and PR is a mainstay in pathological laboratories worldwide. Standardised scoring systems (e.g., percentage of positive tumour nuclei) are in place, facilitating the integration of these markers into routine practice. Recent large multicentre studies have further solidified this, demonstrating that ER and PR expression retain independent prognostic relevance across all molecular subgroups, including the NSMP subgroup (14). Importantly, a refined three-tiered scoring system (0-10%, 20-80%, 90-100%) has been shown to improve prognostication, with high ER expression (90-100%) linked to the highest disease-specific survival rate within the NSMP subgroup (14). For NSMP EC, ER/PR status provides a fundamental stratification: positive expression aligns with a low-risk, hormone-driven pathway, while loss signals a more dedifferentiated, high-risk biology (5).

The clinical utility of ER as a treatment-guiding biomarker in NSMP is now being prospectively tested in the phase III RAINBO NSMP-ORANGE trial (NCT05255653-3). This international, multicentre trial enrols ER-positive NSMP patients (stage II with LVSI or stage III) and randomises them to either pelvic radiotherapy followed by two years of oral progestin or standard chemoradiation. The trial directly addresses whether ER-positive status can identify a subgroup of NSMP patients in whom chemotherapy can be safely omitted—a hypothesis that aligns precisely with the ‘ultra-low-risk’ category proposed in our integrative framework.

However, it is important to note that the prognostic utility of ER may vary in specific clinicopathological contexts. A dedicated study of high-grade (G3) NSMP tumours revealed a high frequency of ER loss (53%), frequent co-occurrence with L1CAM overexpression, and substantial genomic instability. Notably, within this already high-risk cohort, ER status did not provide significant additional prognostic stratification. This suggests that ER status may have limited value for further risk assessment in high-grade NSMP tumours (15). This characteristic defines a uniformly high-risk group where the inherent aggressiveness of the tumour may supersede the prognostic signal of individual markers like ER.

### 2.3 Other molecular markers: ARID1A, CTNNB1, and PIK3CA/PTEN

Mutations in genes such as ARID1A, CTNNB1, and members of the PI3K pathway (PIK3CA, PTEN) are frequent in NSMP EC. However, the prognostic significance of these mutations—such as their predictive accuracy and clinical relevance—remains ambiguous and inconsistent across studies (6). For instance, CTNNB1 exon 3 mutations have been associated with variable prognostic impact across different studies. While some meta-analyses have confirmed a significant association with recurrence, particularly within the NSMP subtype (16), others have reported no significant impact on disease-free survival (17). The PORTEC-4a trial has further incorporated NSMP-CTNNB1 mutated status as an intermediate-risk profile guiding adjuvant brachytherapy decisions (8), underscoring the clinical relevance of this biomarker in NSMP endometrial cancer. Betella and Ribero (2026), in an accompanying commentary, noted that CTNNB1 exon 3 mutations, along with L1CAM overexpression and substantial LVSI, further refine the heterogeneous outcomes observed within the NSMP and MMRd subgroups (18). Evidence regarding ARID1A is limited and presents conflicting results (6, 9). The detection of these alterations typically requires next-generation sequencing, limiting routine diagnostic application. Currently, evidence is insufficient to recommend their use for standard risk stratification in NSMP EC (9), highlighting the distinct and more established value of the protein markers L1CAM and ER/PR which are immediately actionable in the IHC laboratory.

L1CAM and ER/PR IHC provide actionable, cell-intrinsic biological information—that is, data that can directly guide treatment decisions—augmenting standard pathology reports for NSMP EC. These markers help address the question: “What is the inherent aggressive potential of this tumour cell population?” Nevertheless, this information offers a one-dimensional perspective, as it lacks insight into how the tumour interacts with its microenvironment, which is crucial for a comprehensive understanding of tumour behaviour.

## 3 Spatial cancer-immune phenotypes: a functional dimension for diagnosis

The density and spatial distribution of immune cells within the tumour immune microenvironment (TIME) are critical determinants of clinical outcomes. These factors often provide more detailed information about prognosis and treatment response than the mere presence or absence of immune cells, as they reflect the complexity of immune-tumour interactions (19, 20). Spatial cancer-immune phenotypes (SCIs) classify the tumour microenvironment into three main, prognostically distinct patterns based on the spatial relationship between lymphocytes and carcinoma cells (21). These patterns are the immune-inflamed phenotype, characterised by lymphocytes-particularly CD8+ T cells-diffusely infiltrating the tumour epithelium; the immune-excluded phenotype, where immune cells are present but physically restricted to the peritumoral stroma and unable to penetrate the tumour nests; and the immune-desert phenotype, defined by a general paucity of lymphocytes throughout both the tumour parenchyma and stroma.

### 3.1 Definition and diagnostic methodology

The objective classification of SCIs relies on the multiplexed analysis of immune cell markers—such as CD8, CD3, CD68, CD20, and PD-L1—on whole-tissue sections (22, 23). Among the markers used for SCI classification, PD-L1 serves a dual role. First, it functions as a complementary marker to distinguish desert from excluded phenotypes, alongside CD68 and CD20 (21). Second, its expression in NSMP tumours is generally low, consistent with the immune-cold characteristics of this subtype. While PD-L1 positivity in tumour cells has been associated with favourable prognosis in high-risk endometrial cancer, its role as a predictive biomarker for immunotherapy in NSMP remains less defined than in MMRd tumours. Thus, within the current framework, PD-L1 is best viewed as a component of SCI characterisation rather than an independent stratification variable. While traditional haematoxylin and eosin (H&E) assessment of tumour-infiltrating lymphocytes (TILs) provides a preliminary view, it lacks the specificity and spatial resolution to reliably distinguish between the ‘excluded’ and ‘desert’ phenotypes, which have divergent prognostic implications (22). Given these limitations, multiplex immunohistochemistry (mIHC) and immunofluorescence (mIF), coupled with digital pathology and advanced image analysis software, represent the gold standard (23). These tools allow for the simultaneous detection of multiple markers, the precise segmentation of tissue compartments (tumour epithelium versus stroma), and the quantitative analysis of immune cell densities and distances. This transition from subjective visual scoring to quantitative, algorithm-driven classification is essential to ensure the reproducibility necessary for diagnostic implementation (24).

### 3.2 Prognostic validation and diagnostic value in NSMP EC

A seminal study by de Biase et al. (2025) provided the first direct evidence of the strong independent prognostic significance of SCIs specifically within the NSMP subtype (21). While Horeweg et al. (2020) established the prognostic value of quantitative immune cell density across all molecular subtypes (22), de Biase et al. (2025) provided the first direct evidence that the categorical inflamed/excluded/desert trichotomy carries strong independent prognostic significance specifically within the NSMP subtype (21). In their cohort of 86 NSMP ECs (from a total of 213 consecutive endometrial carcinomas), de Biase et al. demonstrated that SCI is a strong prognostic factor specifically within the NSMP subtype. The 3-year recurrence-free survival (RFS) rates were 96.2% (95% CI, 75.7–99.5) for the immune-inflamed phenotype, 83.2% (95% CI, 60.7–93.5) for the immune-desert phenotype, and 40.5% (95% CI, 10.0–70.1) for the immune-excluded phenotype, with a median follow-up of 23 months (range 1–144 months). Crucially, this prognostic stratification remained significant in multivariate analysis. It was particularly pronounced in early-stage disease, precisely where traditional risk assessment is most challenging (21). These findings were independently corroborated by Hattori et al. (2025), who demonstrated across 145 endometrial cancer patients that the non-inflamed (excluded or desert) phenotype was associated with markedly poorer prognosis compared with the inflamed phenotype. Interestingly, this study observed that the “immune-desert” phenotype was particularly common within the NSMP subgroup (24). This observation highlights the heterogeneity of immune responses in NSMP tumours. The variation in findings between studies may reflect cohort differences or methodological nuances, but it collectively underscores the prognostic power of spatial immune analysis and the need for standardised integration with molecular data. Furthermore, specific SCIs correlate with distinct histopathological patterns of invasion, such as the microcystic, elongated, and fragmented (MELF) pattern and tumour budding, linking microarchitectural features of aggressiveness with a dysfunctional, non-inflamed immune contexture (21, 25). This suggests that SCI analysis adds a critical functional layer to morphological diagnosis by addressing whether the host immune response is effectively engaged with the tumour or is spatially or functionally impaired.

The immune-excluded phenotype is of particular interest for immunotherapy, as it represents a potentially modifiable state of immune exclusion that may be amenable to combination strategies aimed at overcoming physical barriers to T cell infiltration. In NSMP EC, which is predominantly MMR-proficient, and microsatellite stable, PD-L1 expression is variable, and the role of immune checkpoint inhibitors remains unclear. However, preliminary evidence suggests that biomarkers of immune exclusion may identify a subset of patients who could benefit from combinations of immunotherapy with anti-angiogenic agents or other modulators of the tumour microenvironment. This represents a critical direction for future translational research in this subtype.

### 3.3 Challenges for implementation as a diagnostic tool

The transition of SCI analysis from a robust research tool to routine diagnostics faces several hurdles. It requires standardised and validated mIHC/mIF protocols, access to digital pathology platforms with image analysis capabilities, as well as the establishment of inter-observer reproducibility for pattern classification (23, 24). Furthermore, efforts are ongoing to define clinically actionable and cost-effective diagnostic algorithms, such as determining which marker panels are essential. Nevertheless, it’s clear and powerful prognostic value—especially its ability to identify the high-risk excluded phenotype, which is otherwise morphologically cryptic—provides a compelling rationale for overcoming these challenges (21). Its integration would represent a move towards a more functional, microenvironment-aware pathological assessment. However, it must be acknowledged that mIHC/mIF platforms remain inaccessible in many routine and lower-resource laboratories owing to high costs, demanding technical requirements, and limited infrastructure, underscoring the need for simplified and cost-effective alternatives to enable broader clinical implementation.

## 4 Building an integrative diagnostic framework: rationale, hypothesis, and challenges

Despite their individual strengths, molecular marker IHC (reflecting cell-intrinsic biology) and SCI analysis (reflecting the host-tumour interaction) currently exist in parallel diagnostic and research streams. Their results are rarely synthesised into a unified pathological interpretation that captures the holistic biology of an NSMP tumour (6, 26).

### 4.1 The state of separation in current practice

The pioneering work by Horeweg et al. (2020) demonstrated that machine learning-based quantification of intraepithelial CD8+ cells improves upon the prognostic utility of molecular classification in early-stage endometrial cancer (22). Our framework differs from and extends this work in three key respects. First, while Horeweg et al. included all molecular subtypes, we focus specifically on the NSMP subtype—the largest and most prognostically heterogeneous group. Second, we operationalise the categorical inflamed/excluded/desert spatial phenotypes rather than a continuous immune cell density measure, aligning with the emerging consensus that spatial distribution—not just density—determines immune functionality. Third, and most importantly, we translate the immune-molecular integration into a discrete risk matrix with explicit links to adjuvant therapy decisions (de-escalation vs. intensification), directly engaging with the clinical decision-making frameworks established by PORTEC-4a and RAINBO NSMP-ORANGE.

In current practice, a biomarker such as L1CAM may be reported as a standalone item indicating either a “positive” or “negative” result, while the complex immune microenvironment remains undescribed in the diagnostic report. While Horeweg et al. (2020) established that quantitative image-based immune profiling can improve upon molecular classification in early-stage endometrial cancer, their study encompassed all molecular subtypes and utilised continuous measures of CD8+ and CD103+ cell density (22). To date, no study has specifically operationalised the categorical inflamed/excluded/desert spatial phenotypes in combination with NSMP-specific IHC markers (L1CAM, ER) to generate a directly actionable risk matrix. This fragmented approach fails to capture the likely synergistic interplay between a tumour’s intrinsic molecular wiring and the immune landscape it actively sculpts, potentially missing the opportunity for superior risk prediction (26).

### 4.2 The biological logic for integration

Tumour biology is not modular. Cell-intrinsic molecular alterations, such as L1CAM overexpression driving invasion and metastasis and ER loss promoting dedifferentiation and stemness, actively shape the tumour immune microenvironment through the secretion of chemokines, cytokines, and other immunomodulatory factors, as well as by altering cell adhesion properties (27, 28). For instance, epithelial-mesenchymal transition (EMT), a process linked to aggressiveness, is known to foster an immunosuppressive and exclusionary microenvironment (28). Therefore, it is biologically plausible that specific molecular profiles are systematically associated with, and may contribute to, characteristic spatial immune architectures. This biological interplay may underlie the profound clinical heterogeneity observed within the NSMP group, which even robust protein markers like L1CAM and ER cannot fully distinguish on their own (6, 9). Preliminary transcriptomic data from Hattori et al. (2025) suggest that in NSMP tumours, type I interferon response genes are enriched in the non-inflamed phenotype, suggesting a potential mechanistic link between specific immune signalling pathways and the formation of immune-excluded or immune-desert microenvironments (24). Investigating this connection is a natural and necessary progression for diagnostic pathology, moving from cataloguing isolated features—i.e., identifying what is present—to understanding their functional relationships—i.e., how they interact.

### 4.3 Proposing an “integrative diagnostic subtyping” hypothesis

Based on this rationale, the integration of these two powerful, complementary prognostic dimensions could yield a more robust and biologically coherent risk stratification system for NSMP EC; this integration forms a testable hypothesis. Consequently, we propose a “Dual-Risk” Integrative Diagnostic Subtyping model, which is conceptualised in **Table 1**.

**Table 1:** Proposed “dual-risk” integrative diagnostic subtyping framework for NSMP endometrial carcinoma.

Molecular risk profile	Spatial immune phenotype	Integrated subtype & risk stratum	Proposed biological/clinical implication
Low-Risk (ER-high, 90-100%)	Inflamed	Integrative Low-Risk	Concordant favourable signals. Excellent prognosis. Candidate for treatment de-escalation (consistent with RAINBO NSMP-ORANGE population).
Low-Risk (ER-high, 90-100%)	Excluded	Discordant (Uncertain)	Molecular-immune conflict. Molecular-risk-first principle applies: manage as Low-Risk but with caution. Priority for further research.
Low-Risk (ER-high, 90-100%)	Desert	Discordant (Uncertain)	Molecular-immune conflict. Molecular-risk-first principle applies: manage as Low-Risk but with caution. Priority for further research.
High-Risk (L1CAM+ ≥10% and/or ER−)	Inflamed	Discordant (Uncertain)	Molecular-immune conflict. Molecular-risk-first principle applies: manage as High-Risk. Priority for further research.
High-Risk (L1CAM+ ≥10% and/or ER−)	Excluded	Integrative High-Risk	Concordant unfavourable signals. Strong rationale for treatment intensification or enrolment in clinical trials.
High-Risk (L1CAM+ ≥10% and/or ER−)	Desert	Integrative High-Risk	Concordant unfavourable signals. Strong rationale for treatment intensification or enrolment in clinical trials.

Additional Notes:.

• Molecular Risk Definitions: Low-Risk = ER-high (90-100% positive tumour nuclei by IHC); High-Risk = L1CAM+ (≥10% tumour cells) and/or ER− (<1% or <10% tumour nuclei, per study-specific thresholds). ER serves as the primary anchor; L1CAM acts as a risk modifier.

• Discordant Combinations: Classified provisionally by the molecular-risk-first principle, reflecting the stronger evidence base for ER and L1CAM in current guidelines and trials. Such cases are recommended for management per current clinical guidelines pending further validation.

• SCI Definitions: Inflamed = dense lymphocyte infiltration within tumour epithelium; Excluded = lymphocytes restricted to peritumoral stroma; Desert = scant lymphocytes throughout both epithelial and stromal compartments.

• Modifying Factors: CTNNB1 mutational status (e.g., NSMP-CTNNB1 mutated in PORTEC−4a) is not included as an independent stratification variable but should be considered as a modifying factor in the interpretation of Low-Risk profiles.

• High-Grade NSMP: G3 NSMP tumours behave uniformly aggressively regardless of ER or L1CAM status and constitute a separate uniformly high-risk group.

EC, Endometrial carcinoma; ER, Oestrogen receptor; IHC, Immunohistochemistry; L1CAM, L1 cell adhesion molecule; NSMP, No Specific Molecular Profile; SCI, Spatial cancer-immune phenotype.

#### 4.3.1 Molecular risk strata

The molecular dimension of our framework comprises two primary strata, anchored by ER and L1CAM—the two biomarkers with the strongest evidence base in NSMP endometrial cancer.

Low-Risk is defined by ER-high expression (90-100% positive tumour nuclei by IHC). This threshold is derived from the refined three-tiered scoring system validated by Timmermans et al. (2026) and aligns with the ER-positive eligibility criteria of the RAINBO NSMP-ORANGE trial.

High-Risk is defined by L1CAM overexpression (≥10% tumour cells by IHC) and/or ER-negative status (<1% or <10% tumour nuclei, per study-specific thresholds). This definition integrates the PORTEC-4a criteria for L1CAM-associated unfavourable risk with the ESGO-ESTRO-ESP guidelines for ER-negative high-risk NSMP.

The priority between these two markers follows a principled hierarchy: ER status serves as the primary anchor, while L1CAM acts as a risk modifier. In ER-positive tumours, L1CAM overexpression does not automatically reclassify the tumour as High-Risk; instead, it flags a discordant profile requiring caution. In ER-negative tumours, L1CAM positivity reinforces the High-Risk classification and supports the strongest treatment escalation. This hierarchy reflects the more established role of ER in current clinical guidelines and trials, while preserving the documented prognostic value of L1CAM.

#### 4.3.2 Spatial immune phenotypes

The spatial immune dimension comprises three categorical phenotypes as defined in Section 3, based on the distribution of immune cells (predominantly CD8+ T lymphocytes) relative to tumour epithelium:

- Immune-Inflamed: dense lymphocytic infiltration within the tumour epithelium
- Immune-Excluded: lymphocytes confined to the peritumoral stroma, absent from tumour nests
- Immune-Desert: scant lymphocytes throughout both epithelial and stromal compartments

These phenotypes were independently validated as prognostically distinct in NSMP by de Biase et al. (2025) (21)and corroborated in the broader endometrial cancer cohort by Hattori et al. (2025) (24).

#### 4.3.3 Integration matrix and classification logic

The intersection of the two molecular strata and three immune phenotypes yields six potential combinations, as summarised in **Table 1**. These are consolidated into four clinical categories:

1. Integrative Low-Risk: Low-Risk molecular (ER-high) with Immune-Inflamed phenotype. The concordant favourable signals identify patients with an excellent prognosis, aligning with the RAINBO NSMP-ORANGE de-escalation population.
2. Integrative High-Risk: High-Risk molecular (L1CAM+ and/or ER−) with Immune-Excluded or Immune-Desert phenotype. The concordant unfavourable signals identify patients at highest risk of recurrence, warranting treatment intensification or enrolment in clinical trials.
3. Discordant (Uncertain): This category comprises two scenarios:

- Low-Risk molecular (ER-high) with Immune-Excluded or Immune-Desert
- High-Risk molecular (L1CAM+ and/or ER−) with Immune-Inflamed

In these combinations, molecular and immune signals point in opposite directions. We apply a provisional molecular-risk-first principle, consistent with the stronger evidence base for ER and L1CAM in current clinical guidelines and trials. However, we explicitly acknowledge that the prognosis of these discordant cases remains uncertain and that they constitute a priority area for future research.

4. High-Grade NSMP: As previously reported by Stelloo et al. (2016), high-grade (G3) NSMP tumours—regardless of ER or L1CAM status—behave uniformly aggressively (15). We therefore classify such tumours as a separate, uniformly high-risk group, for which further stratification by these markers may be of limited additional value.

#### 4.3.4 Classification of discordant combinations

For the two concordant combinations—molecular low-risk (ER-high) with immune-inflamed, and molecular high-risk (L1CAM-positive or ER-negative) with immune-excluded—the framework assigns clear prognostic categories (integrative low-risk and integrative high-risk, respectively). For discordant combinations—where molecular and immune signals point in opposite directions (e.g., ER-high with immune-excluded, or L1CAM-positive with immune-inflamed)—the available evidence does not permit a definitive classification.

In the absence of direct data comparing the relative prognostic weights of these two dimensions, we propose a provisional working rule: molecular risk takes precedence over immune phenotype when the two are discordant. This rule is based on the stronger evidence base for ER and L1CAM in NSMP, including their incorporation into the 2025 ESGO–ESTRO–ESP guidelines (ER) and the PORTEC-4a trial (L1CAM). Accordingly, any L1CAM-positive or ER-negative tumour, regardless of SCI, would be managed as high-risk; any ER-high tumour with a discordant immune phenotype would be assigned to an intermediate/uncertain category pending further data.

We emphasise that this is a testable hypothesis, not a validated rule. The discordant categories are precisely where future research is most needed, and they represent priority areas for prospective validation in cohorts such as the RAINBO NSMP-ORANGE trial or PORTEC-4a translational sub studies. Until such data become available, we recommend that patients with discordant profiles be managed according to current clinical guidelines, with the understanding that their optimal treatment remains undefined.

#### 4.3.5 Modifying role of CTNNB1

Although CTNNB1 mutational status is not included as an independent stratification variable in the current version of our framework, we acknowledge its prognostic relevance within the NSMP subtype. The PORTEC-4a trial classified NSMP-CTNNB1 mutated tumours as intermediate risk (vaginal brachytherapy), whereas NSMP-CTNNB1 wild-type tumours were classified as favourable (observation) (8). This evidence suggests that CTNNB1 mutation may modulate the prognosis of ER-high tumours, adding a note of caution to an otherwise Low-Risk classification. We therefore recommend that CTNNB1 status be considered as a modifying factor in the interpretation of Low-Risk profiles, particularly when L1CAM and immune phenotypes are also discordant.

#### 4.3.6 Evidence base and limitations

The evidence underlying each cell of the matrix is derived from the following sources: ER-related risk strata are supported by the ESGO-ESTRO-ESP guidelines (4) and the RAINBO NSMP-ORANGE trial; L1CAM-related risk strata are supported by the PORTEC-4a trial (8) and multiple meta-analyses (2, 9); SCI prognostic associations are supported by de Biase et al. (2025) (21)and Hattori et al. (2025) (24). The integration of these dimensions, and the classification rules proposed for discordant cases, represent a testable hypothesis that requires prospective validation. The provisional nature of these rules, particularly for discordant profiles, is explicitly acknowledged, and we prioritise this area for future research.

### 4.4 Diagnostic challenges on the path to implementation

Translating this integrative framework into reproducible clinical practice requires confronting fundamental pre-analytical and analytical standardisation challenges—an issue highlighted by initiatives such as the NCI Biospecimen Evidence-Based Practices program (29)-alongside additional hurdles in evidence generation, clinical utility assessment, and reporting:

1. Technical and Analytical Standardisation: Harmonising pre-analytical (tissue processing), analytical (mIHC/digital analysis protocols), and post-analytical (scoring algorithms, cut-offs) workflows across laboratories is paramount.
2. Evidence Generation: Large-scale, multi-institutional retrospective and prospective validation studies are needed to confirm the incremental prognostic value of the integrated model over single-marker approaches and to refine risk group definitions. The RAINBO NSMP-ORANGE trial provides a unique opportunity to validate key aspects of our proposed framework. First, it prospectively tests the hypothesis that ER-positive status alone can guide treatment de-escalation—a hypothesis that our framework would refine by asking whether the addition of spatial immune phenotyping can further identify which ER-positive patients (inflamed vs. excluded/desert) derive greatest benefit from de-escalation, or which might still benefit from chemotherapy despite ER positivity. Second, translational sub studies of the NSMP-ORANGE biospecimen cohort could test whether SCI adds incremental prognostic value beyond ER status, directly informing whether our proposed ‘dual-risk’ matrix should be incorporated into future iterations of the RAINBO trial design.
3. Clinical Utility and Cost-Effectiveness: Studies must demonstrate that making treatment decisions based on this integrated classification leads to improved patient outcomes (e.g., reduces recurrence in high-risk groups or safely reduces overtreatment in low-risk groups) in a cost-effective manner.
4. Reporting and Integration into Workflows: Developing structured, clear, synoptic reporting templates to communicate the integrated diagnosis effectively to clinicians is essential for adoption.

Beyond adjuvant treatment decisions, the proposed integrative framework may also have implications for surgical management, although this remains speculative. If spatial immune phenotyping can identify patients with indolent tumour biology (e.g., ER-high/inflamed), one could hypothesise that such patients might be candidates for less extensive surgical staging, analogous to the trend toward sentinel lymph node biopsy in low-risk endometrial cancer. Conversely, patients with adverse molecular-immune profiles (e.g., L1CAM+/excluded) might warrant comprehensive surgical staging given their higher risk of occult metastasis. However, we emphasise that this hypothesis requires prospective validation and is not currently supported by direct clinical evidence. Pending such data, surgical decisions should continue to follow current guidelines based on established histopathological risk factors.

## 5 Conclusion and perspectives: implications for future diagnostic pathology

The management of NSMP endometrial carcinoma stands at a crossroads. While traditional histopathology provides an indispensable foundation, the integration of deeper biological insights is essential for more biologically informed clinical decision-making (3, 6, 7). Molecular markers like L1CAM and ER/PR, alongside SCIs, offer powerful and biologically grounded prognostic tools. However, their full potential remains untapped due to a siloed approach to diagnosis and reporting. The clinical relevance of the molecular component of our framework—particularly L1CAM and ER—is now supported by the PORTEC-4a trial, which has demonstrated that L1CAM can safely guide adjuvant radiotherapy decisions, and the RAINBO NSMP-ORANGE trial, which is prospectively testing ER-guided de-escalation. The next logical step is to evaluate whether spatial immune phenotyping adds incremental value to this established molecular risk stratification.

The integrative framework proposed herein is designed to augment the WHO classification by providing a clinically actionable “integrated risk profile.” By systematically combining accessible molecular markers (L1CAM, ER/PR) with spatial immune architecture, its primary utility lies in directly informing adjuvant therapy decisions. It aims to resolve the current dilemma by identifying: (1) ‘ultra-low-risk’ patients (e.g., ER-high/immune-inflamed) for whom therapy might be safely de-escalated; and (2) ‘ultra-high-risk’ patients (e.g., L1CAM-positive/immune-excluded) who may merit treatment intensification or novel therapies. This directly addresses the heterogeneity unresolved by current methods.

This review advocates for a conceptual shift toward integrative diagnostic subtyping. The proposed “dual-risk” model (**Table 1**) is a testable hypothesis firmly rooted in established tumour biology principles and supported by multiple independent lines of prognostic evidence (9, 11, 21, 24, 26). If validated, this model could contribute to diagnostic pathology by enabling a more nuanced, functionally informed risk assessment that directly tackles the core challenge of NSMP EC.

The path forward requires a concerted, collaborative effort. In the short term, priority should be given to multi-centre retrospective studies using standardised protocols to test the core associations. In the medium term, the development of consensus diagnostic guidelines and reporting standards will be crucial. In the long term, leveraging the ongoing digital transformation of pathology will be key to making this comprehensive, integrated profiling efficient, scalable, and accessible in routine practice (23, 24). By embracing this integrative approach, diagnostic pathology can move beyond its traditional descriptive role to play a central part in guiding personalised, risk-adapted therapy, with the ultimate goal of improving patient outcomes by ensuring the right treatment is delivered to the right patient.

## 6 Critical view

This review differs from existing literature by systematically integrating two previously siloed prognostic dimensions—protein-level molecular markers (L1CAM, ER/PR) and spatially resolved cancer-immune phenotypes—into a unified diagnostic framework specifically for NSMP endometrial carcinoma. Prior studies have independently validated the prognostic value of L1CAM, ER/PR, and SCIs (2, 9, 21, 24), but none have proposed a testable combinatorial model that directly links molecular and immune profiles to adjuvant therapy decisions.

Several knowledge gaps remain. First, standardisation of multiplex immunohistochemistry (mIHC) and digital pathology algorithms for SCI classification is lacking, which limits the reproducibility and clinical implementation of this approach (23, 24). Second, the biological mechanisms linking specific molecular alterations—such as L1CAM overexpression or ER loss—to distinct spatial immune patterns (e.g., immune-excluded versus immune-desert) are poorly understood, although epithelial-mesenchymal transition has been implicated (21, 28). Third, prospective validation of the proposed “dual-risk” hypothesis in multi-centre cohorts is absent; current evidence is derived from retrospective analyses with heterogeneous methodologies.

The framework presented here provides a starting point for risk stratification that moves beyond single biomarkers. By identifying ultra-low-risk (e.g., ER-high/immune-inflamed) and ultra-high-risk (e.g., L1CAM-positive/immune-excluded) phenotypes, it offers clinically testable categories that could inform personalised management, including treatment de-escalation or intensification. However, the clinical utility of this model requires rigorous validation before adoption.

The RAINBO NSMP-ORANGE trial represents a key opportunity for prospective validation of the ER-based component of our framework. Its biospecimen cohort could be leveraged to test whether spatial immune phenotyping adds independent prognostic value to ER status in NSMP, potentially informing a refined stratification within the trial’s ER-positive population.

Future research should prioritise: (i) development of standardised mIHC/mIF protocols and reference materials for SCI classification to enable inter-laboratory reproducibility; (ii) large-scale retrospective (e.g., PORTEC cohort) and prospective studies to validate the incremental prognostic value of the integrative model over conventional histomorphology; (iii) mechanistic investigations into crosstalk between molecular drivers and immune exclusion, potentially leveraging single-cell spatial transcriptomics; and (iv) cost-effectiveness analyses to support routine diagnostic adoption. Until these gaps are addressed, the framework remains a testable hypothesis rather than a ready-to-use clinical tool.
